# Use of Botanical Ingredients: Nice Opportunities to Avoid Premature Oxidation of NABLABs by Increasing Their ORAC Values Strongly Impacted by Dealcoholization or Pasteurization

**DOI:** 10.3390/molecules29102370

**Published:** 2024-05-17

**Authors:** Margaux Simon, Hubert Kageruka, Sonia Collin

**Affiliations:** Unité de Brasserie et des Industries Alimentaires, Louvain Institute of Biomolecular Science and Technology (LIBST), Faculté des Bioingénieurs, Université Catholique de Louvain, Croix du Sud, 2 Box L7.05.07, B-1348 Louvain-la-Neuve, Belgium; margaux.simon@uclouvain.be (M.S.); hubertk2@gmail.com (H.K.)

**Keywords:** antioxidant power, ORAC, NABLABs, pasteurization, dark malts, *Vernonia amygdalina*

## Abstract

Even when fresh, non-alcoholic, and low-alcoholic beers (NABLABs) exhibit significant staling defects due to premature oxidation. In this study, the antioxidant power of eleven fresh commercial NABLABs was assessed by means of three different assays: the oxygen radical absorbance capacity (ORAC), the linoleic acid-induced oxidation (TINH), and the indicator time test (ITT). Only the first two assays, both involving radicalar degradations initiated by AAPH, were found to correlate with each other. NABLABs displayed lower ORAC values than conventional beers (on average, 6127 μmol eq. Trolox/L), except for three samples made with special-colored malts or dry-hopped. Dealcoholization was the step with the greatest impact on the ORAC value (up to a 95% loss) and on flavan-3-ols, sotolon, and polyfunctional thiols, while pasteurization strongly affected color, TBA, and Strecker aldehydes. ORAC assays applied to hop, alternative cereals, and various botanical ingredients indicated that mashing with red sorghum, dry hopping/spicing, and wood maturation could bring the antioxidant power of a NABLAB close to those of conventional beers. With an ORAC value not reached by any other tested botanical ingredient (5234 µmol eq. Trolox/g), African *Vernonia amygdalina* leaves (traditionally used for Rwandan Ikigage beers) emerged here as the best candidate.

## 1. Introduction

Slowing down aroma staling to extend a beer’s shelf life remains one of the major challenges for the brewing industry [1,2]. Oxidation is often the primary contributor to flavor instability [3]. Much effort has been devoted to minimizing oxygen uptake during brewing and packaging [1,4,5,6]. Increasing antioxidant concentrations can also inhibit the effects of oxygen by scavenging reactive oxygen species or free radicals, chelating transition metal ions (copper and iron), decomposing peroxides, etc. [3,7,8,9,10,11,12]. A large number of assays have been published in the literature for measuring antioxidant activity, some of them taking into account more specific properties [7,11,13,14]. As many oxidative mechanisms can occur in a complex matrix, it could be advised to combine several assays for beer investigations.

**Figure 1:**
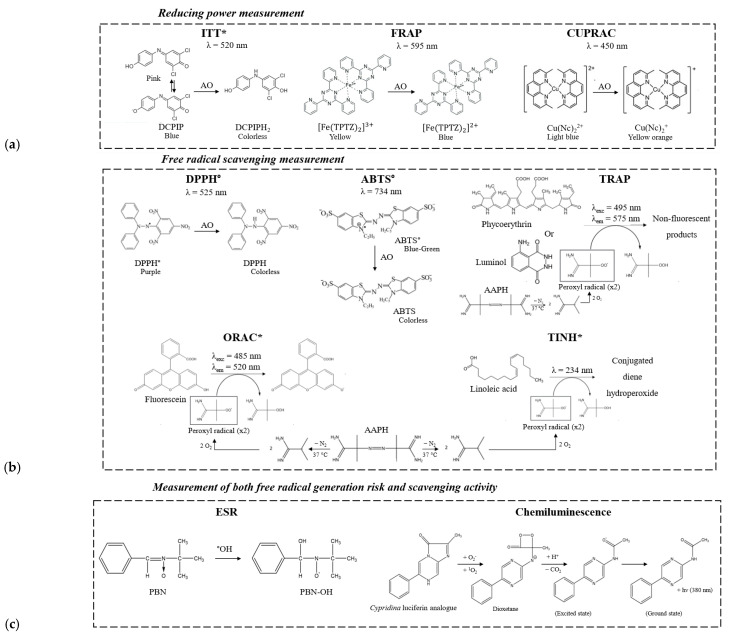
Examples of antioxidant activity assays used in the brewing field. * Selected in the present study.

Interest was first concentrated on the oxidoreduction reactions (colorimetric or electrochemical methods), which could inform about the reducing power of wort and beer (e.g., 2,6-dichlorophenolindophenol in the indicator time test (ITT) [15], iron dipyridyl complex [16], redox potential [17], FRAP (ferric reducing antioxidant parameter) [9], and CUPRAC (cupric reducing antioxidant capacity) [18]) (Figure 1a). Nowadays, it is accepted that reactive oxygen species (ROS) such as hydroxyl radical HO° and superoxide radical O_2_°^−^ are agents causing beer damage. Therefore, most assays prefer measuring the free radical scavenging activity of the medium (e.g., DPPH° reducing activity [19], ABTS° decolorization assay [20], superoxide scavenging activity in the xanthine/xanthine oxidase system [21], and the scavenging of the hydroxyl radical in deoxyribose [21] or leucomethylene blue [22] assays) (Figure 1b).

In many cases (including the ORAC and TINH assays), peroxyl radicals are artificially created by the thermal decomposition of 2,2′-azobis(2-methylpropionamidine) dihydrochloride (AAPH). A great advantage of the ORAC method (in which the radicalar degradation of fluorescein is easily monitored by UV fluorescence) is its very high sensitivity [23]. Moreover, ORAC values have been determined for a wide range of food matrices [24], for example, 5693 μmol eq. Trolox/100 g for red wine, 9645 μmol eq. Trolox/100 g for hazelnut, or 20,823 μmol eq. Trolox/100 g for black chocolate. Another sensitive method in which the oxidation kinetic of an aqueous dispersion of linoleic acid is followed was described by Liégeois et al. [25] as more representative of what happens in a dispersed lipid matrix such as wort or beer. Products resulting from this peroxidation are the conjugated diene hydroperoxides, which absorb at 234 nm. When antioxidants are present in beer, oxidation is delayed, and the resulting inhibition period (TINH) is determined. In order to assess also the pro-oxidant activity of the medium (iron cations, etc.), radicals in beer can still be monitored by electron spin resonance (ESR) or luminescence analysis (Figure 1c) [26,27,28].

In conventional beers, both endogenous and exogenous antioxidants can play a crucial role in delaying or preventing oxidative damage [2,29]. Natural antioxidants originate mainly from barley malt and kettle hopping [11,30]. Both contribute to beer polyphenols [31,32,33], while only special malts bring significant amounts of reductones and melanoidins [3,8,11,34,35]. Therefore, in most cases, the total polyphenol content of a beer correlates directly (R^2^ = 0.8) with its antioxidant activity (contribution of 55–88%) [1,8,31,36]. Unfortunately, oxidation products derived from polyphenols can also negatively affect color and colloidal stability [8,37,38]. During fermentation, yeast also produces antioxidants, mainly sulfites (through the conversion of sulfates, methionine, or cysteine) and glutathione [3,8,26,39]. Moreover, sulfites and ascorbic acid can be added to the bottle as exogenous antioxidants. In addition to their antimicrobial activity, sulfites consume bottled oxygen, thus protecting other antioxidant fractions [5,8,35]. Other antioxidants present in beer at very low levels include carotenoids and tocopherols [31,32,40], saponarin, and hordatines A-C [8,41]. Some additional antioxidants may come from dry hopping [37], spices/herbs (e.g., hibiscus, juniper, lemon balm, etc. [42,43]), fruits (e.g., cherry juice and goji berry [44,45]), flavorings and colorings [7], or alternative raw materials (e.g., sorghum and buckwheat [46,47]).

Whatever the process used (dealcoholization, cold contact, special yeast, etc.), non-alcoholic and low-alcoholic beers (NABLABs, NAB ≤ 0.5% ABV and LAB 0.5–1.2% ABV in most European countries) are usually brewed at lower original extract levels, leading to lower total polyphenol contents (75–366 mg GAE/L versus 875 mg GAE/L for bock beer) [30,48,49] and lower melanoidin levels (0.58 mg/L versus 1.49 mg/L for dark beer) [11,34,35]. Furthermore, the dealcoholization and stronger pasteurization (at least 50 UP versus 15 UP for conventional beers [50,51]) usually applied to NABLABs can also degrade the antioxidant capacity of the medium. Dealcoholized beers have been found to display about a third of the antioxidant power of bock beers (1525 versus 4663 µmol Fe^2+^/L as determined by FRAP assay) [31].

**Figure 2:**
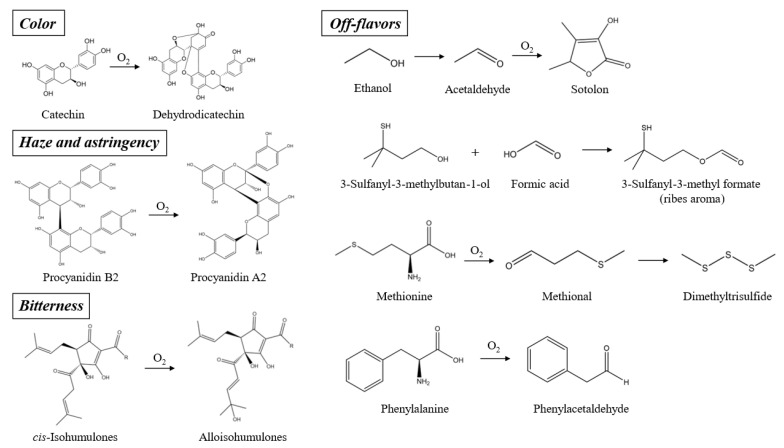
Oxidation of flavan-3-ols, isohumulones, and precursors of odorants, impacting color, haze, astringency, bitterness, and flavor.

Unsurprisingly, fresh NABLABs often suffer from premature oxidation (Figure 2). This has an impact on both bitterness and astringency, by enhancing isohumulone and flavan-3-ol oxidation [38]. *trans*-Isohumulones are known to be the most degraded fraction, given their propensity to be converted to tricyclohumols. For *cis*-isohumulones, oxidative degradation to alloisohumulones is the main concern [52,53]. For flavan-3-ols, it is now recognized that the oxidation of catechins to dehydrodicatechins increases color, while oligomer oxidation leads to colloidal instability and astringency [38]. The odorants sotolon (curry), phenylacetaldehyde (floral, honey), methional (boiled potato), and dimethyltrisulfide (onion) have recently been detected at higher levels in such beers [54].

The aim of the present work was to compare the antioxidant power of eleven commercial NABLABs with conventional beers. ORAC, TINH, and ITT values were related to levels of various previously quantitated beer constituents. The impacts of both dealcoholization and pasteurization on the ORAC value and aromas were further assessed on two pilot samples. Lastly, to determine the feasibility of increasing NABLAB antioxidant activity, an ORAC assay was applied to sorghum, spices, wood, and other promising botanical extracts in order to calculate the amount required to reach in NABLABs an ORAC value similar to that of conventional beers.

## 2. Results and Discussion

### 2.1. ORAC Values of Fresh NABLABs and Relationship to Color, Phenols, and Bitterness

As depicted in Table 1, almost all fresh NABLABs, whatever the process used, showed significantly lower antioxidant power (on average 6127 μmol eq. Trolox/L) than a conventional lager (10,171 μmol eq. Trolox/L), a dry-hopped beer (11,456 μmol eq. Trolox/L), or a Trappist brown ale (12,332 μmol eq. Trolox/L). The relatively low densities of the worts commonly employed in NABLAB production (around 5 °P) most probably limit their polyphenol content (43–150 mg/L, Table 1). Moreover, intrinsic antioxidants can be altered by dealcoholization and pasteurization, procedures often applied to NABLABs.

**Figure 3:**
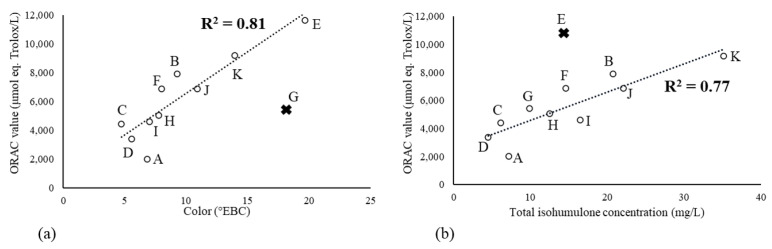
Correlations for fresh NABLABs between ORAC value and (**a**) color or (**b**) total isohumulone concentration (letter: name of sample and cross: sample exclude from correlation).

Interestingly, beers E and K exhibited the highest values (11,637 and 9193 μmol eq. Trolox/L, respectively), likely due to the use of special/colored malts known to contain antioxidant melanoidins [11]. As shown in Figure 3a, a correlation was observed between the ORAC value and color (R^2^ = 0.81 if the red fruit wheat beer G was not included).

Beer B also reached a slightly higher value (7906 μmol eq. Trolox/L) because of its dry hopping process. Hop is known to show a 30 times greater intrinsic antioxidant capacity than pale malt [25,55], thanks to its very high level of polyphenols [37,55,56]. The total polyphenol content, as already shown by other studies [8,41,57], appeared to contribute most to the antioxidant power of each beer (42–100%; Table 1), with a major proportion attributed to flavan-3-ols (catechin ORAC value = 11.2 μmol eq. Trolox/μmol) [1,31,41] and phenolic amino acids (2.1 and 1.0 μmol eq. Trolox/μmol for tryptophan and tyrosine, respectively) [41,58]. No relationship was found here with polyphenols. 

Surprisingly, we also observed a correlation between the ORAC value and the isohumulone content (R^2^ = 0.77 without beer E whose cold contact process provided better protection against oxidation, Figure 3b). There should be no direct causative link here, as isohumulones (produced by isomerization in the boiling kettle from hop humulones) showed almost no antioxidant activity (ORAC value = 0.1 μmol eq. Trolox/μmol; Table 1). Yet, the level of bitter compounds depends on the amount of hop used, as does the level of polyphenols (which indirectly elucidates this correlation). 

### 2.2. Comparison of the ORAC Assay with Two Other Antioxidant Assays Used on NABLABs

**Figure 4:**
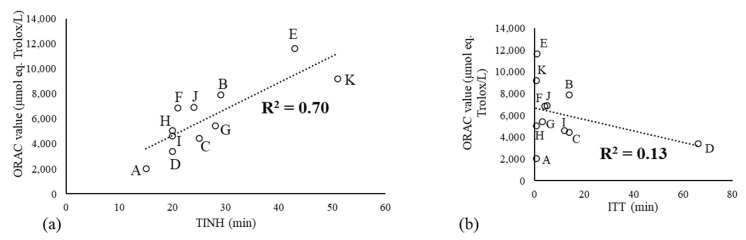
Correlations for fresh NABLABs between ORAC values and (**a**) TINH or (**b**) ITT values (letter: name of sample).

In parallel with the ORAC assay, two additional antioxidant power measurements were applied to the eleven NABLABs: TINH, which also involves a radicalar reaction initiated by AAPH (linoleic acid used here as substrate instead of fluorescein), and the ITT test, which involves a simpler redox reaction (Figure 1). Whatever the method used, the antioxidant power of NABLABs remained poor (Table 1). Not surprisingly, a correlation was found only between the ORAC and TINH values (R^2^ = 0.70, Figure 4a). The non-radicalar ITT test showed no correlation with the ORAC value (R^2^ = 0.13, Figure 4b).

### 2.3. Impact of NABLAB Dealcoholization and Pasteurization on ORAC Values, Thermal Indicators, Bitter Compounds, Phenols, and Aromas

Two pilot blond beers (A and B; initially at 5.6% and 4.7% ethanol (*v*/*v*), respectively) were subjected to vacuum distillation (industrial NABLAB production operating at 35–40 °C and 100 mbar) and tunnel pasteurization (50 UP for A and 90 UP for B). Antioxidant activity (ORAC), thermal indicators (color and TBA), bitter compounds, phenols, stale odorants, and hoppy polyfunctional thiols were determined before dealcoholization (BD), after dealcoholization (AD), and after pasteurization (AP) (Table 2).

The dealcoholization of either sample led to a huge ORAC value decrease (loss of up to 59% for sample A and 95% for sample B). The antioxidant activity decreased further through pasteurization, leading to only 1042 and 291 μmol eq. Trolox/L (which is even lower than the values found in the eleven investigated commercial NABLABs, probably due to the lower-scale experiments). The data of previous chemiluminescence studies confirm an increase in the level of oxidation in conventional beers (a five times higher OH-radical signal intensity) after pasteurization [59,60,61,62], whereas, surprisingly, Lund et al. found an increased antioxidant capacity, likely due to formation of Maillard compounds [2,59].

As previously reported by Callemien et al. [63], total polyphenol values are not good indicators of intrinsic oxidative changes in flavan-3-ol chemical structures (loss of only 10 mg/L after dealcoholization in sample A). On the other hand, catechin and procyanidin B3 dropped strongly from 3.2 to 1.3 mg/L and from 1.8 to 0.9 mg/L in sample B, respectively, clearly evidencing the occurrence of oxidation through both dealcoholization [64] and pasteurization.

Our two thermal indicators showed that dealcoholization had little impact on heat-related reactions, compared to pasteurization: Specifically, an increase of 2.5–3 °EBC and 17–22 TBA was observed between AD and AP, whereas color slightly decreased during dealcoholization [64]. A higher degree of pasteurization (50–90 UP) and, consequently, a greater thermal load are required for NABLABs. Colored compounds resulting from Maillard reactions are logically formed at this step.

Oxidation of *cis-* and *trans*-isohumulones occurred during both dealcoholization and pasteurization (a loss of *cis*-isohumulones up to 2.6 mg/L, in sample B), in parallel with the synthesis of their oxidative degradation products such as alloisohumulones [53,65], reaching 0.2–0.4 mg/L isohumulone equivalents (other by-products, including tricyclohumols, were not determined here).

Among the stale odorants often detected in NABLABs even when fresh, sotolon was found at 0.9–1.4 µg/L after pasteurization (values significantly above its sensory threshold of 0.8 µg/L in both samples). In both cases, dealcoholization already slightly increased the amount of this oxidative aroma. On the other hand, only pasteurization caused a marked increase in methional and phenylacetaldehyde (oxygen not required for thermal Strecker degradation).

In contrast, most fresh hoppy/citrus polyfunctional thiols were lost upon dealcoholization (3SHol dropped from 4.3 µg/L to 0.3 µg/L in sample A). One should note, however, that some can be added at the time of pasteurization, most probably coming from cysteinyl precursors.

### 2.4. Potential to Increase NABLAB ORAC Values by Using Sorghum, Vernonia amygdalina, Spices, or Wood Chips

In order to assess how to enhance the NABLAB antioxidant capacity, ORAC values of alternative cereals, spices, other botanical ingredients, and wood chips were determined, and for each, the quantity needed to achieve the antioxidant power of a conventional beer in NABLABs was calculated (Table 3). For comparison, ORAC values of ascorbic acid and potassium metabisulfite (KMS) (antioxidants often used in breweries; Table 3) show that extravagant spiking would be required, both with KMS (386 g/hL = 3860 mg/L for a maximum of 20 mg/L allowed) and ascorbic acid (87 g/hL= 870 mg/L; compared to the 30–50 mg/L amount usually added).

As shown here with the Citra hop sample (one of the varieties richest in flavanoids, along with Saaz [56]), dry hopping above 850 g/hL would effectively boost the ORAC value of a NABLAB into the target range (this was only partially achieved in the Belgian dry-hopped commercial beer B, with its 7906 μmol eq. Trolox/L). In the United States, hop is often used up to 500–1000 g/hL (2000 g/hL even reached for NEIPAs).

Interestingly, *Vernonia amygdalina* leaves (used in some traditional Rwandan sorghum beers known as Ikigage [66]) exhibited the highest antioxidant power (5234 μmol eq. Trolox/g). Only 99 g/hL would be needed (if no loss occurs through the process) to reach the antioxidant capacity of a conventional beer. Malted red sorghum (855 μmol eq. Trolox/g) should also make it possible to substantially increase the antioxidant activity of NABLABs (only 5–10% of barley malt should be here replaced by sorghum malt). Red sorghum is known to contain exceptional amounts of flavan-4-ols, 3-deoxyanthocyanidins, flavones, and flavan-3-ols (up to hexamers) [67]. As an additional advantage, this cereal also contains little beta-amylase, the enzyme that brewers avoid in NABLAB wort mashing (lower maltose content). Among the spices/herbs investigated here, the best candidates were cinnamon, ginger, and orange peel (ORAC values of 907, 721 and 510 μmol eq. Trolox/g, respectively), although more than 500 g/hL would be required during boiling or fermentation/maturation to reach the antioxidant activity of a conventional beer (probably too much in terms of flavor; generally added from 5 to 225 g/hL, depending on the type of spice). With their 1036 and 980 μmol eq. Trolox/g, acacia and oak chips, possibly added during maturation, also appear as reasonable candidates (500 g/hL often used by brewers for wood-aged beers [68]).

## 3. Materials and Methods

### 3.1. Chemicals

Acetic acid, acetone, acetonitrile, ammonia solution 28–30%, anhydrous sodium sulfate, citric acid monohydrate, dichloromethane, dipotassium hydrogen phosphate trihydrate, ethanol absolute 99%, formic acid, hydrochloric acid 37%, methanol, potassium dihydrogen phosphate, potassium hydroxide, sodium chloride, and sodium hydroxide were purchased from VWR International (Leuven, Belgium). 2-Acetylthiophene, ammonium iron (III) citrate 16%, 2,2′-azobis(2-methylpropionamidine) dihydrochloride (AAPH), Amberlite XAD-2 resin, boric acid, carboxymethylcellulose sodium salt, >98% L-cysteine hydrochloride monohydrate, (±)-catechin hydrate, decane, 2,6-dichlorophenolindophenol, 6 mL Discovery Ag-ion SPE tube, (−)-epicatechin, fluorescein sodium salt, linoleic acid 99%, methional, nonadecane, phenylacetaldehyde, Sephadex LH-20 resin, sotolon, 3-sulfanylhexan-1-ol (3SHol), 3-sulfanylhexyl acetate (3SHA), 6-sulfanylhexan-1-ol, 2-thiobarbituric acid, titriplex III, Trolox^®^ ((±)-6-hydroxy-2,5,7,8-tetramethylchromane-2-carboxylic acid), tryptophan and tyrosine were purchased from Sigma-Aldrich (Overijse, Belgium). Isohumulone standard was purchased from Labor Veritas Co. (Zürich, Switzerland). Procyanidin B3 and (+)-taxifolin standards were from Extrasynthèse (Genay, France). AccQ•Tag Ultra Reagent derivatization (6-aminoquinolyl-N-hydroxysuccinimidyl carbamate, AQC), AccQ•Tag Ultra Eluent I, AccQ•Tag Ultra Eluent II, and AccQ•Tag Ultra borate buffer were purchased from Waters Corporation (Milford, CT, USA). Milli-Q water was used (Millipore, Bedford, MA, USA).

### 3.2. Samples

Eleven commercial NABLABs were investigated: Star Light (A; special blond), Energibajer (B; dry-hopped), Pico Bello (C; dry-hopped), Leopold 7 Road Trip (D; sour beer), Palm N.A. (E; amber), Maes 0.0% (F; lager), Hoegaarden rosée 0.0% (G; red fruit white beer), Carlsberg 0.0% (H; lager), Jupiler 0.0% (I; lager), Leffe Blonde 0.0% (J; abbey beer), and Brugse Sport Zot alcoholvrij (K; special blond). The beers, either received from brewers or bought at Belgian markets (freshly released), were analyzed in duplicate. Pilot samples of two beers (A and B), taken both before dealcoholization (BD) and after dealcoholization (AD), as well as after pasteurization (AP; 50 UP for A and 90 UP for B), were provided by AB-Solutions (Courcelles, Belgium) and brewers. Spices/herbs (coriander, orange peel, cardamom, licorice, cinnamon, ginger, and hibiscus) were supplied by Fagron (Nazareth, Belgium), and wood chips (oak, mulberry, and acacia) were obtained from Wilhelm Eder GmbH (Bad Dürkheim, Germany). *Vernonia amygdalina* leaves and flowers were harvested in Rwanda.

### 3.3. Standard Analyses on NABLABs and Pilot Samples

Prior to analysis, beers were degassed by shaking and filtered through paper filters (MN 614 ¼ Macherey-Nagel, Düren, Germany). The alcohol content was determined with DM4500 apparatus (Anton Paar GmbH, Graz, Austria), and color was analyzed by means of Analytica-EBC 9.2. and 9.6 [69]. TBA (thiobarbituric Acid Index) was analyzed according to the ASBC method Wort 21 [70].

### 3.4. Antioxidant Assays

The solid matrices (1 g), after grinding, were first extracted with 10 mL of a mixture of acetone/water/acetic acid (70:29.5:0.5, *v*/*v*/*v*) and centrifuged for 15 min at 3000 rpm. The extraction and ORAC analysis were conducted in duplicate.

#### 3.4.1. ORAC Values of NABLABs, Chemical Standards, Pilot Samples, and Botanical Extracts

The ORAC procedure with fluorescein as a “fluorescent probe” (substrate) was carried out at 37 °C on an automated 96 white opaque wells plate reader (Synergy HT, Bio-Tek, Winooski, VT, USA) working at an excitation wavelength of 485 nm and an emission wavelength of 520 nm. The reaction was started by the thermal decomposition of AAPH. Working solutions of fluorescein (55 nM), AAPH (153 µM), and Trolox^®^ (200 µM) were freshly prepared in phosphate buffer (75 mM, pH 7.4) from stock solutions stored under refrigeration conditions. In each well, 250 µL of fluorescein and 25 µL of the sample (suitable dilution to prepare in advance), blank, or standard (Trolox^®^ at 8, 16, 24, 32, and 40 µM) were added. The plate was then heated to 37 °C for 10 min prior to the addition of 25 µL of AAPH. The fluorescence was measured immediately and every minute for 50 min. The ORAC values, expressed as µmol Trolox equivalents/g fresh mass (or /L for liquid extracts), were calculated with the following equation: ORAC value = (AUC_sample_ − AUC_blank_)/(AUC_Trolox_ − AUC_blank_) × Trolox^®^ concentration (µM) × dilution factor with AUC = area under fluorescence curve.

#### 3.4.2. TINH Values of NABLABs

The antioxidant activity was determined as the inhibition times of linoleic acid oxidation induced in an aqueous solution by the free radical initiator AAPH [25]. Briefly, 30 μL of the 16 mM linoleic acid dispersion (in borate buffer 0.05 M, pH 9) was added to the UV cuvette containing 2.81 mL of phosphate buffer (0.05 M, pH 7.4), prethermostated at 40 °C. The oxidation reaction was initiated at 37 °C under air by the addition of 150 µL of 40 mM AAPH solution (in phosphate buffer). Oxidation was carried out in the presence of 10 µL of beer samples. In the assay without antioxidants, lipid oxidation was measured in the presence of the same level of methanol. The oxidation rate at 37 °C was monitored by recording the increase in absorption at 234 nm caused by conjugated diene hydroperoxides. A Shimadzu UV–visible 240 spectrophotometer (Antwerp, Belgium) equipped with an automatic sample positioner allowed for the analysis of six samples every minute. The measurements were run in duplicate against the buffer and compared with a separate AAPH-free control to check for any spontaneous oxidation (AAPH has a relatively high absorbance below 260 nm, which changes as the compound decomposes). Therefore, its absorbance measured in a separate cuvette in the absence of linoleic acid was subtracted from each experimental point. The inhibition time (TINH) was estimated with Microsoft Excel (Microsoft 365 version 2404) and Geogebra Classic software (version 6.0.841.0) as the point of intersection between the tangents to the inhibition and propagation phase curves.

#### 3.4.3. ITT Values of NABLABs

The ITT assay measures the discoloration time of an indicator, 2,6-dichlorophenolindophenol (DCPIP, 1450 mg/L), which is blue in its oxidized form and turns colorless when reduced by antioxidants in beer. First, four samples were prepared: 50 mL water with pH adjusted to that of beer + 250 µL DCPIP (comparator solution); 10 mL beer + 250 µL DCPIP (indicator solution); beer; and distilled water. Subsequently, 10 mL of each solution was placed in a Hellige’s comparator. The comparator solution, with a dilution resembling 80% DCPIP discoloration, was introduced into the left-hand lens of the comparator. This was positioned in front of the tube containing the beer to simulate the turbidity present in the indicator solution on the right. The DCPIP indicator, influenced by the antioxidants in beer, was gradually reduced and discolored. The time required for the indicator solution to reach the same discoloration as the comparator solution was then measured.

### 3.5. Analyses of Bitter Compounds in NABLABs and Pilot Samples by High-Performance Liquid Chromatography–Ultraviolet Detection (HPLC-UV)

Beer samples were degassed by shaking and diluted twice in methanol. After 15 min, the mixture was filtered through a Chromafil polyester filter (0.45 µm, Macherey-Nagel, Düren, Germany). Separation was performed on two C8 columns in tandem: the Zorbax Eclipse XDB-C8 150 × 4.6 mm, 5 µm, and the Zorbax Eclipse XDB-C8 150 × 4.6 mm, 3.6 µm (Agilent Technologies, Santa Clara, CA, USA), using the binary solvent system of Analytica EBC method 9.47 [69] with A: methanol; B: 1% aqueous citric acid solution (pH 7.0)–acetonitrile (70:30, *v*/*v*). Gradient elution was as follows: 15% A for 5 min, increasing A to 80% over 25 min, and 80% A for 3 min. The column temperature was kept at 35 °C, the flow rate at 1.0 mL/min, and the injection volume was 50 µL. Chromatograms were recorded throughout elution with the Empower software version 2002 (Build 1154, Waters Corporation, Milford, CT, USA). The retention time and absorption spectrum of isohumulones were obtained by injection of standards. An absorbance wavelength of 270 nm was chosen for isohumulone and alloisohumulone quantitation (absorbance spectrum λ_max_ = 228 and 280 nm [65]). Quantitation was performed using a single-point calibration, as suggested by the EBC method 9.47 [69].

### 3.6. Phenols Quantitation in NABLABs and Pilot Samples

#### 3.6.1. Total Polyphenol Measurement

Total polyphenol content was analyzed according to Analytica EBC method 9.11 [69].

#### 3.6.2. Catechin, Epicatechin, and Procyanidin B3 Determination by HPLC-UV

Beer flavan-3-ols (catechin, epicatechin, and procyanidin B3) were extracted on Sephadex LH-20 resin. Briefly, 3 g of resin packed in a 12 mL filtration tube SPE with a polyethylene frit was preconditioned for 4 h with methanol–water (30:70, *v*/*v*). The flux was set at 0.5 mL/min. After loading 50 mL of degassed beer containing 2.8 mg/L of IST ((+)-taxifolin), the column was washed with 40 mL of methanol–water (30:70, *v*/*v*). Flavan-3-ols were recovered with 70 mL of acetone–water (70:30, *v*/*v*). The eluate was concentrated to dryness by vacuum rotary evaporation (35 °C) and dissolved in 2 mL of acetonitrile–water (30:70, *v*/*v*). The extracts were kept at −80 °C prior to analysis.

An Agilent 1200 Series liquid chromatography system (Agilent Technologies, Santa Clara, CA, USA) equipped with an autosampler, a quaternary pump, and a UV detector set at 280 nm was used. A 150 × 2.1 mm, 3 µm C18 Prevail column (HICHROM, Deerfield, IL, USA) was used at a flow rate of 0.2 mL/min. Chromatographic separation was obtained using a multilinear gradient of water containing 0.1% formic acid (A) and acetonitrile containing 0.1% formic acid (B). Gradient elution was 97–91% A, 0–5 min; 91–85% A, 5–30 min; 85–67% A, 30–60 min; 67–0% A, 60–70 min; 0–97% A, 70–75 min; and then return to the initial conditions for 15 min. Ten microliters of beer extract were injected into the column kept at 25 °C. Chromatograms were recorded throughout elution using ChemStation software (version B.04.03). Quantitation was achieved using the calibration curves (relative to the IST).

#### 3.6.3. Tryptophan and Tyrosine Quantitation by Ultra-Performance Liquid Chromatography–UV Detection (UPLC-UV)

Briefly, 10 μL of a degassed beer sample, filtered through a Chromafil polyester filter (0.22 μm, Macherey-Nagel, Düren, Germany), was mixed with 70 μL of borate buffer and 20 μL of AQC derivatization reagent. The mixture was then heated at 55 °C for 10 min. An ACQUITY UPLC liquid chromatography system (Waters Corporation, Milford, CT, USA), equipped with a degasser, an autosampler, an oven, a quaternary pump, and a UV detector set at 210 nm was used. Separation was carried out on ACQUITY UPLC BEH C18 (100 × 2.1 mm, 1.7 μm column—Waters Corporation) at a flow rate of 0.65 mL/min, with a mixture of A (Eluent I), B (10% Eluent II in water), C (water), or D (Eluent II). Gradient elution was as follows: 0.0–0.29 min, 10–9.9% A and 90–90.1% C; 0.29–5.49 min, 9.9–9% A, 0–80% B, and 90.1–11% C; 5.49–7.10 min, 9–8% A, 80–15.6% B, 11–57.9% C, and 0–18.5% D; 7.10–7.30 min, 8% A, 15.6% B, 57.9% C, and 18.5% D; 7.30–7.69 min, 8–7.8% A, 15.6–0% B, 57.9–70.9% C, and 18.5–21.3% D; 7.69–7.99 min, 7.8–4% A, 70.9–36.3% C and 21.3–59.7% D; 7.99–8.59 min, 4% A, 36.3% C, and 59.7% D; 8.59–8.68 min, 4–10% A, 36.3–90% C, and 59.7–0% D; 8.68–10.20 min, 10% A and 90% C. One microliter of mixture was injected into the column kept at 42 °C. Chromatograms were recorded throughout elution using Empower 2 software. Tryptophan and tyrosine identification was performed by the injection of a commercial mixture of standards. Quantification was achieved using the calibration curves.

### 3.7. Pilot Sample Aroma Extraction

#### 3.7.1. XAD-2 Resin Extraction of Sotolon, Methional, and Phenylacetaldehyde, and Quantification by Gas Chromatography-Electron-Impact Mass Spectrometry (GC-MS)

For apolar compounds extraction, 2 g of Amberlite XAD-2 resin was added to a 50 mL degassed beer sample containing 150 µL of 2-acetylthiophene (IST, 8 mg/L, final beer concentration = 24 µg/L). For sotolon extraction, the pH of the beer was adjusted to 11.5 with sodium hydroxide. The two mixtures were shaken at 200 rpm for 2 h. The content of the flask was then transferred into a glass column (60 × 1 cm, i.d.). For apolar aromas, the resin was first rinsed with 4 × 50 mL of Milli-Q water to eliminate sugar and other water-soluble substances. They were then eluted with 2 × 20 mL of bidistilled dichloromethane. For sotolon, the eluate from the resin and the first 50 mL of resin washing water were mixed before bringing the pH to 3.0 with hydrochloric acid. This aqueous phase was extracted three times with 40 mL of bidistilled dichloromethane (10 min, 2500 rpm). All extracts were then dried with anhydrous sodium sulfate, and 25 µL of decane or nonadecane (for sotolon) solution (250 mg/L) was added as EST before concentration reached 500 µL in a Danish– Kuderna at 45 °C (total concentration factor = 100). The final extracts were stored at −80 °C until analysis by GC-MS.

One microliter of each aroma extract was analyzed with an Agilent Technologies 7890 NB Gas Chromatograph System equipped with a splitless injector (250 °C). Apolar compounds were separated using a wall-coated open tubular (WCOT) apolar capillary column (CP-Sil 5 CB, 50 m × 0.32 mm, 1.2 µm). The oven temperature was programmed to rise from 36 to 85 °C at 20 °C/min, then to 145 °C at 1 °C/min, and to 250 °C at 3 °C/min, and then held for 30 min. Sotolon was analyzed with a WCOT polar capillary column (FFAP CB, 25 m × 0.32 mm, 0.3 µm). The oven temperature was programmed to rise from 36 to 85 °C at 20 °C/min, then to 145 °C at 1 °C/min, followed by 160 °C at 3 °C/min, and 230 °C at 3 °C/min, and then held for 30 min. The carrier gas was helium, and the pressure was set at 100 kPa (50 kPa for sotolon). The column was connected to a quadrupole mass spectrometer (Agilent 5977 MSD) operating in single-ion monitoring (SIM) mode with electron ionization at 70 eV. The following *m/z* values were monitored: 111 and 126 for 2-acetylthiophene, 71 and 85 for decane and nonadecane, 91 and 120 for phenylacetaldehyde, 104 and 76 for methional, and 83 and 128 for sotolon. Chromatograms were recorded throughout elution (Agilent OpenLab software version 2.1 used). Calibration curves (with areas relative to IST) were constructed for each compound, and the following equation was used for quantitation of compound A: concentration of A (in µg/L) = IST concentration (in µg/L) × (A area/IST area) × (IST response coefficient/A response coefficient). The IST relative recovery factor was set at 1 for all compounds.

#### 3.7.2. Ag Selective Extraction of Polyfunctional Thiols, and Quantification by Gas Chromatography–Pulsed-Flame Photometric Detection (GC-PFPD)

Briefly, 2 µg/L 6-sulfanylhexan-1-ol was added as IST to 150 mL beer, which was then saturated with NaCl and stirred with 50 mL dichloromethane for 15 min. The mixture was centrifuged at 4500 rpm for 15 min. The recovered organic phase was loaded onto a Discovery Ag-ion SPE cartridge conditioned beforehand with 10 mL dichloromethane. The cartridge was rinsed with 10 mL dichloromethane, then with 20 mL acetonitrile, and finally with 10 mL ultrapure water (reversed cartridge in this last case). Free thiols were released from the Ag cartridge by percolating 20 mL washed cysteine solution (4 × 20 mL dichloromethane for washing 215 mg cysteine in 20 mL water). The eluent was extracted twice with bidistilled dichloromethane (5 mL for 5 min and 10 mL for 10 min). The resulting organic phase was dried on anhydrous sodium sulfate and concentrated to 250 µL in a Danish–Kuderna distillation apparatus and to 70 µL on a Dufton column at 45 °C. 2-Acetylthiophene was added as EST (0.5 mL at 200 µg/L added before concentration).

One microliter of free thiol extract was analyzed with an Agilent 6890N gas chromatograph equipped with a splitless injector maintained at 250 °C. Compounds were analyzed with WCOT apolar capillary column (CP-Sil 5 CB, 50 m × 0.32 mm, 1.2 µm). The helium pressure was set at 90 kPa. The oven temperature was programmed to increase from 36 to 85 °C at 20 °C/min, then to 145 °C at 1 °C/min, and finally to 220 °C at 3 °C/min, and was held for 30 min. The column was connected to the OI Analytical PFPD detector (model 5380, combustor internal diameter: 2 mm). The following parameters were selected for the PFPD detector: temperature, 250 °C; voltage, 600 V; gate width, 18 ms; gate delay, 6 ms; trigger level, 400 mV; pulse frequency, 3.33 Hz. PFPD chromatograms were recorded throughout elution. The ChemStation software was used to process the resulting data. For all thiols, the IST-relative recovery factor was set at 1 (experimental values from 0.8 to 1.2, determined beforehand by standard addition). The following equation was used for the quantitation of the commercially available standards 3SHol, and 3SHA: thiol concentration (in µg/L) = IST concentration (in µg/L) × (thiol area/IST area) × (IST weight response coefficient/thiol weight response coefficient). For the commercially unavailable standards, 3-sulfanyl-4-methylpentanol (3S4MPol), and 3-sulfanyl-4-methylpentyl acetate (3S4MPA), the good equimolarity of the PFPD detector enabled us to set the IST-relative molar response coefficients at 1 and to apply only the corrective molar weight ratio: thiol concentration (in µg/L) = IST concentration (in µg/L) × (thiol area/IST area) × (thiol molar weight /IST molar weight).

### 3.8. Statistical Analyses

All analytical measurements were carried out in duplicate. Multiple comparisons of means were performed with Student–Newman–Keuls tests (JMP Program). Values sharing no common letter are significantly different (*p* < 0.05).

## 4. Conclusions

Commercial NABLABs displayed only half the antioxidant capacity of conventional beers, except for three samples made with special-colored malts or dry-hopped. Surprisingly, a correlation (R^2^ = 0.77) was observed between the ORAC value and the isohumulone content, even though isohumulones showed almost no antioxidant activity. Phenolic compounds contributed most to the antioxidant power of NABLABs. Dealcoholization had a strong impact on the ORAC value, flavan-3-ols, sotolon, and hop polyfunctional thiols, while pasteurization mainly affected color, TBA, and Strecker aldehydes. Red sorghum mashing, dry hopping/spicing, and wood maturation could reasonably increase the antioxidant power of a NABLAB to a level approaching those of conventional beers. Interestingly, *Vernonia amygdalina* leaves emerged here as the best candidate, with an ORAC value (5234 μmol eq. Trolox/g) not reached by any other tested botanical ingredient. NABLAB production trials should be carried out to confirm these findings.

## Figures and Tables

**Table 1:** Ethanol, color, isohumulones, phenols, and antioxidant activity (ORAC, TINH, and ITT values) determined for fresh NABLABs. Values in parentheses give the contribution (%) of each fraction to the measured ORAC value, determined on the basis of analyses performed on four reference standards (0.1, 11.2, 2.1, and 1.0 μmol eq. Trolox/μmol for isohumulone, catechin, tryptophan, and tyrosine).

Beer Samples	Ethanol (% *v*/*v*)	Color (°EBC)	Isohumulones (mg/L)		Phenols (mg/L)						Antioxidant Activity		
			*cis-*	*trans-*	Total polyphenols	(+)-Catechin	(−)-Epicatechin	Procyanidin B3	Tryptophan	Tyrosine	ORAC value (μmol eq. Trolox/L)	TINH (min)	ITT (min)
NABLABs			**Special yeasts**										
A	0.5	6.9	5.5 ^e^ (<0.1)	1.7 ^e,f^ (<0.1)	43 ^f^ (82)	1.0 ^f^ (1.9)	0.3 ^f^ (0.6)	1.1 ^c^ (1.1)	2.7 ^g^ (1.3)	4.8 ^i^ (1.3)	2014 ^g^	15 ^f^	0.7 ^d^
B	0.3	9.3	16.0 ^c^ (<0.1)	4.7 ^b^ (<0.1)	124 ^d^ (60)	1.5 ^e^ (0.7)	1.0 ^b^ (0.5)	2.0 ^b^ (0.5)	15.9 ^d^ (2.0)	25.5 ^f^ (1.7)	7906 ^c^	29 ^c^	14 ^b^
C	0.2	4.7	5.8 ^e^ (<0.1)	0.3 ^g^ (<0.1)	135 ^d^ (>100)	3.5 ^a^ (3.0)	1.2 ^a^ (1.0)	1.0 ^c^ (0.4)	15.4 ^e^ (3.5)	26.0 ^e^ (3.2)	4428 ^e^	25 ^d^	14 ^b^
			**Limited fermentation or cold contact**										
D	0.8	5.6	4.3 ^e^ (<0.1)	0.2 ^g^ (<0.1)	56 ^f^ (64)	2.1 ^b^ (2.3)	0.9 ^c^ (1.0)	1.2 ^c^ (0.7)	nd	12.4 ^h^ (2.0)	3382 ^f^	20 ^e^	66 ^a^
E	0.1	19.7	10.9 ^d^ (<0.1)	3.6 ^c^ (<0.1)	149 ^d^ (49)	1.1 ^f^ (0.4)	0.8 ^c,d^ (0.3)	0.7 ^c^ (0.1)	16.0 ^d^ (1.4)	35.0 ^c^ (1.6)	11,637 ^a^	43 ^b^	0.8 ^d^
			**Vacuum dealcoholization**										
F	<0.1	8.0	12.0 ^d^ (<0.1)	2.6 ^d,e^ (<0.1)	84 ^e^ (47)	1.4 ^e^ (0.8)	0.8 ^c,d^ (0.5)	0.9 ^c^ (0.3)	17.6 ^b^ (2.6)	33.0 ^d^ (2.6)	6865 ^d^	21 ^e^	4 ^c^
G	<0.1	17.8	9.4 ^d^ (<0.1)	0.5 ^g^ (<0.1)	171 ^c^ (>100)	1.6 ^d,e^ (1.1)	0.6 ^e^ (0.4)	0.9 ^c^ (0.3)	0.7 ^h^ (0.1)	3.4 ^j^ (0.3)	5420 ^e^	28 ^c^	3 ^c^
H	0.1	7.8	10.7 ^d^ (<0.1)	1.8 ^e,f^ (<0.1)	68 ^e,f^ (52)	1.8 ^c,d^ (1.4)	0.3 ^f^ (0.2)	1.0 ^c^ (0.4)	16.5 ^c^ (3.3)	38.0 ^a^ (4.1)	5047 ^e^	20 ^e^	0.7 ^d^
I	<0.1	7.0	15.3 ^c^ (<0.1)	1.2 ^f,g^ (<0.1)	50 ^f^ (42)	0.9 ^f^ (0.7)	0.3 ^f^ (0.2)	0.7 ^c^ (0.3)	8.6 ^f^ (1.9)	24.1 ^g^ (2.8)	4621 ^e^	20 ^e^	12 ^b^
J	<0.1	10.9	18.9 ^b^ (<0.1)	3.2 ^c,d^ (<0.1)	269 ^b^ (>100)	1.9 ^b,c^ (1.1)	0.7 ^d^ (0.4)	1.1 ^c^ (0.3)	nd	nd	6890 ^d^	24 ^d^	5 ^c^
			**Filtration dealcoholization**										
K	0.5	13.9	28.9 ^a^ (<0.1)	6.2 ^a^ (<0.1)	304 ^a^ (>100)	3.6 ^a^ (1.5)	1.0 ^b^ (0.4)	2.6 ^a^ (0.5)	19.0 ^a^ (2.1)	35.5 ^b^ (2.1)	9193 ^b^	51 ^a^	0.7 ^d^
Conventional beers													
Lager	5.2	5.7									10,171 ^b^		
Dry-hopped	6.0	18.2									11,456 ^a^		
Trappist brown beer	9.0	60.0									12,332 ^a^		

Within a column, values with different letters are significantly different (*p* < 0.05) according to the Student–Newman–Keuls test; nd: not detected in sample by UPLC.

**Table 2:** Antioxidant activity, color, thermal load, bitter compounds, phenols, and aromas in two pilot samples before dealcoholization (BD), after dealcoholization (AD), and after pasteurization (AP).

	Sample A			Sample B		
	BD	AD	AP	BD	AD	AP
Antioxidant Activity						
ORAC value (μmol eq. Trolox/L)	8238 ^a^	3372 ^b^	1042 ^c^	7204 ^a^	355 ^b^	291 ^b^
Thermal indicators						
Color (°EBC)	9.0	7.0	9.5	6.5	5.5	8.5
TBA	35 ^c^	43 ^b^	60 ^a^	12 ^c^	14 ^b^	36 ^a^
Bitter compounds						
Alloisohumulones (mg/L eq. isohumulones)	0.2 ^a^	0.4 ^a^	0.6 ^a^	0.1 ^b^	0.2 ^a,b^	0.3 ^a^
*cis*-Isohumulones (mg/L)	9.3 ^a^	8.6 ^a,b^	7.6 ^b^	11.4 ^a^	10.0 ^b^	8.8 ^c^
*trans*-Isohumulones (mg/L)	6.0 ^a^	5.2 ^a,b^	3.5 ^b^	6.0 ^a^	5.1 ^b^	4.8 ^b^
Phenols (mg/L)						
Total polyphenols	144 ^a^	134 ^a^	148 ^a^	154 ^a^	89 ^b^	107 ^a^
Catechin	2.0 ^a^	1.2 ^b^	0.5 ^c^	3.2 ^a^	1.5 ^b^	1.3 ^b^
Epicatechin	1.0 ^a^	0.6 ^a^	0.5 ^a^	1.4 ^a^	0.8 ^a^	0.6 ^a^
Procyanidin B3	2.0 ^a^	1.4 ^a^	1.4 ^a^	1.8 ^a^	1.2 ^a^	0.9 ^a^
Stale odorants and pleasant polyfunctional thiols (μg/L)						
Sotolon (thr. = 0.8 μg/L)	0.2 ^c^	0.6 ^b^	0.9 ^a^	0.1 ^c^	0.3 ^b^	1.4 ^a^
Methional (thr. = 0.5 μg/L)	0.5 ^b^	0.5 ^b^	1.3 ^a^	0.7 ^b^	0.6 ^b^	2.5 ^a^
Phenylacetaldehyde (thr. = 5.4 μg/L)	7.0 ^b^	8.1 ^b^	28.4 ^a^	7.2 ^b^	5.9 ^b^	10.4 ^a^
3SHol (thr. = 0.055 μg/L)	4.3 ^a^	0.3 ^b^	nd	nd	nd	nd
3SHA (thr. = 0.005 μg/L)	nq	nd	nd	0.3 ^a^	0.1 ^b^	0.1 ^b^
3S4MPol (thr. = 0.07 μg/L)	0.3 ^a^	nd	0.3 ^b^	0.7 ^a^	0.4 ^b^	0.3 ^b^
3S4MPA (thr. = 0.16 μg/L)	0.9 ^a^	nd	0.5 ^b^	0.7 ^a^	nd	nd

thr. = perception threshold, nd = not detected in sample, nq = not quantifiable; within a line, values with different letters are significantly different (*p* < 0.05) according to the Student–Newman–Keuls test.

**Table 3:** ORAC values of brewing antioxidants, alternative cereals, botanical ingredients, spices, and wood chips, and amounts required to achieve the antioxidant power of a conventional beer.

	ORAC Value (μmol eq. Trolox/g)	Amount Required (g/hL Beer for 100% Recovery) to Bring the ORAC Value of a NABLAB (on Average 6127 μmol eq. Trolox/L) to the Antioxidant Power of a Conventional Beer (on Average 11,320 μmol eq. Trolox/L)
Common brewing antioxidants		
Ascorbic acid	5982 ^a^	87
Potassium metabisulfite	1344 ^c,d^	386
Non-conventional cereals		
Unmalted white sorghum	24 ^k^	21,638
Unmalted red sorghum	390 ^h,i,j,k^	1332
Rwandan traditional malted red sorghum	855 ^d,e,f,g,h^	607
Hops		
Citra T-90 pellets	615 ^e,f,g,h,i,j^	844
Saaz T-90 pellets	1101 ^c,d,e^	472
Spices/herbs and other botanical ingredients		
Coriander	273 ^i,j,k^	1902
Orange peel	510 ^f,g,h,i,j,k^	1018
Cardamom	56 ^k^	9273
Licorice	212 ^i,j,k^	2450
Cinnamon	907 ^d,e,f,g,h^	573
Ginger	721 ^e,f,g,h,i^	720
Hibiscus	477 ^g,h,i,j,k^	1089
*Vernonia amygdalina* leaves	5234 ^b^	99
*Vernonia amygdalina* flowers	1457 ^c^	356
Wood chips		
Oak	980 ^c,d,e,f,g^	530
Acacia	1036 ^c,d,e,f^	501
Mulberry	148 ^j,k^	3509

Within a column, values with different letters are significantly different (*p* < 0.05) according to the Student–Newman–Keuls test.

## Data Availability

Data are contained within the article.

## Abbreviations

NABLABs: non-alcoholic and low-alcoholic beers, ABV: alcohol by volume, HPLC: high-performance liquid chromatography, UV: ultraviolet detection, GC: gas chromatography, PFPD: pulsed flame photometric detection, MS: mass spectrometry, IST: internal standard, EST: external standard, ORAC: oxygen radical absorbance capacity, TINH: inhibition time, ITT: indicator time test, AAPH: 2,2′-azobis(2-methylpropionamidine) dihydrochloride, TBA: thiobarbituric acid index, GAE: gallic acid equivalent, UP: pasteurization unit, 3SHol: 3-sulfanylhexan-1-ol, 3SHA: 3-sulfanylhexyl acetate, 3S4MPol: 3-sulfanyl-4-methylpentanol, 3S4MPA: 3-sulfanyl-4-methylpentyl acetate, BD: before dealcoholization, AD: after dealcoholization, AP: after pasteurization, DCPIP: 2,6-dichlorophenolindophenol, SPE: solid-phase extraction.
